# Comparative Evaluation of Depth of Penetration of AH Plus® Sealer With Different Ratios of Pachymic Acid Using a Confocal Scanning Electron Microscope: An In Vitro Study

**DOI:** 10.7759/cureus.110586

**Published:** 2026-06-10

**Authors:** Gayathri Esakkimuthu, Vinaykumar G, Vinoo Subramaniam Ramachandran, Sibi Swamy, Abinaya K, Anitha K

**Affiliations:** 1 Conservative Dentistry and Endodontics, RVS (Rathinavel Subramaniam) Dental College and Hospital, Coimbatore, IND; 2 Dentistry, RVS (Rathinavel Subramaniam) Dental College and Hospital, Coimbatore, IND

**Keywords:** ah plus sealer, confocal scanning electron microscope, depth of penetration, mandibular premolar, pachymic acid, tubular penetration

## Abstract

Introduction: Modification with pachymic acid (PA) has been seen to reduce the cytotoxicity of freshly mixed AH Plus^®^ sealer (Dentsply Sirona Inc., Charlotte, North Carolina, United States). The efficacy of the sealer is enhanced by its effect of tubular penetration. The aim of this study was to compare the efficacy of AH Plus sealer penetration with the addition of 0.75% PA in the ratios of 10:1 and 5:1 using a confocal scanning electron microscope.

Materials and methods: A total of 30 single-rooted mandibular premolar teeth with a single canal were collected and stored in distilled water. All 30 teeth were prepared to a master apical size of 30/0.09 using ProTaper rotary nickel-titanium files (Dentsply Sirona Inc.). The teeth were randomly divided into three groups based on the sealer used for obturation: (i) Group A: AH Plus sealer, (ii) Group B: AH Plus sealer with a 10:1 ratio of 0.75% PA, and (iii) Group C: AH Plus sealer with a 5:1 ratio of 0.75% PA. The obturated teeth were sectioned horizontally at three different levels from the root apex and were studied under a confocal electron microscope. Data were statistically analyzed using ANOVA.

Results: Sealer penetration was highest in Group A, measuring 1640.80 ± 4.5 µm, 985.39 ± 3.2 µm, and 387.91 ± 1.9 µm at the coronal, middle, and apical thirds, respectively (p = 0.03). Group B showed penetration depths of 910.04 ± 1.4 µm, 1007.62 ± 1.9 µm, and 635.95 ± 2.8 µm, while Group C demonstrated 823.54 ± 3.7 µm, 334.03 ± 4.6 µm, and 423.92 ± 2.7 µm at the respective root levels. Overall, Group A (unmodified AH Plus) exhibited the greatest sealer penetration.

Conclusion: In all regions, AH plus sealer with the addition of 10:1 and 5:1 of 0.75% PA showed lesser penetration than that by the AH Plus sealer alone.

## Introduction

A fluid-tight, three-dimensional (3D) seal is the core objective of a successful root canal treatment [1]. Root canal sealers play a vital role in filling the irregularities between the core material, gutta-percha, and the dentinal walls [2]. Among the various groups of sealers available commercially, an epoxy resin-based sealer, AH Plus® (Dentsply Sirona Inc., Charlotte, North Carolina, United States), stands out and is a touchstone material due to its outstanding chemical adherence, simplicity of handling, prolonged operation, and sealing efficacy [3].

Despite being more advantageous, evidence confirms that AH Plus sealer relates to severe cytotoxicity immediately after mixing and may persist for several hours. Postoperative discomfort or pain often arises when AH Plus sealer is not confined within the root canal space and comes in close proximity to the periodontal tissues. This issue is mainly attributed to the depletion of glutathione, the release of formaldehyde, and the presence of bisphenol A glycidyl ether, a mutagenic component that often results in periapical inflammation [4,5].

Since the initial implementation of AH Plus sealer in clinical practice, its composition has been constantly exploited with the addition of various materials to improve its antimicrobial properties. Besides all the synthetic materials, the ingestion of indigenous phytochemicals has been experimented with so that we can use their mechanical effects with fewer side effects. Pachymic acid (PA) is one such phytochemical, a triterpenoid obtained from the fruiting bodies of the *Fomitopsis nigra *mushroom, said to possess anti-inflammatory effects [6]. Evidence has proven that the cytotoxicity of AH Plus sealer decreases upon incorporation of PA when tested on L929 mouse fibroblast cells [7,8]. Preethi et al. have proven that the incorporation of PA did not affect the physicochemical properties of the sealer; rather, it increased the anti-inflammatory effects of the AH Plus sealer [5].

There is a clear correlation between sealer penetration depth and the success of the endodontic treatment. The depth of sealer penetration depends on various physical and chemical properties [9]. Recent studies have shown that the addition of PA influences AH Plus sealer’s flow, and the physicochemical properties of this modified sealer appear to be within the ISO standards [6]. However, this improved depth of penetration has not been investigated. Thus, the purpose of this in vitro investigation is to evaluate the depth of penetration of AH Plus when modified with different ratios of 0.75% PA.

## Materials and methods

This was an in vitro study at the Department of Conservative Dentistry and Endodontics, RVS Dental College and Hospital, Coimbatore, Tamil Nadu, India.

Sample preparation

A total of 30 single-rooted mandibular premolars extracted for orthodontic purposes were selected and stored in normal saline. Buccolingual and mesiodistal radiographs were used to confirm the single canal. Teeth that had more than one canal, fractures, cavities, restorations, or endodontic treatment were not included. 

We performed the decoronation of teeth to a conventional length of 14 mm. Using a #10 K file (Mani, Inc., Utsunomiya, Japan), the patency of the root was determined. A radiograph was done to confirm that the working length was one millimeter short of the apex. In compliance with the manufacturer's specifications, up to F3 (30/0.09) as the master apical size, we constructed canals utilizing the ProTaper Gold rotary system (Dentsply Sirona Inc.) with an endomotor (X-Smart; Dentsply Sirona Inc.). We performed copious irrigation using saline and 3% sodium hypochlorite (NaOCl) during each instrument change. 

Preparation of PA-modified AH plus sealer

We followed the manufacturer's instructions while mixing traditional AH Plus sealer. Next, we mixed 0.75% PA in 0.1 ml (Bio Corporals Co., Ltd., Chennai, Tamil Nadu, India) with 0.9 ml of AH Plus sealer to obtain a 10:1 ratio of AHP10. Similarly, we mixed 0.2 ml of PA with 0.8 ml of AD Plus sealer to obtain a 5:1 ratio of AHP5 [3]

Obturation protocol

After cleaning and shaping, all the samples were randomly divided into three groups based on the sealer used for obturation: (i) Group A: AH Plus sealer (n=10), (ii) Group B: AH Plus sealer with 10:1 ratio of 0.75 % PA (AHP10) (n=10), and (iii) Group C: AH Plus sealer with 5:1 ratio of 0.75% PA (AHP5) (n=10). We irrigated the canals with 2mL of 17% ethylenediaminetetraacetic acid (EDTA) for one minute before obturation in order to eliminate the smear layer. This was followed by a final rinse with 5mL of saline. Afterward, we used absorbent points to dry the canals. The sealer was then combined with 0.1% isothiocyanate fluorescent rhodamine dye to enable fluorescence under confocal laser microscopy. Following the manufacturer's directions, the dye and sealer were combined to create a smooth, uniform paste. The canal walls were uniformly coated with sealers and obturated using a standardized master cone.

Tooth sectioning 

**Figure 1:**
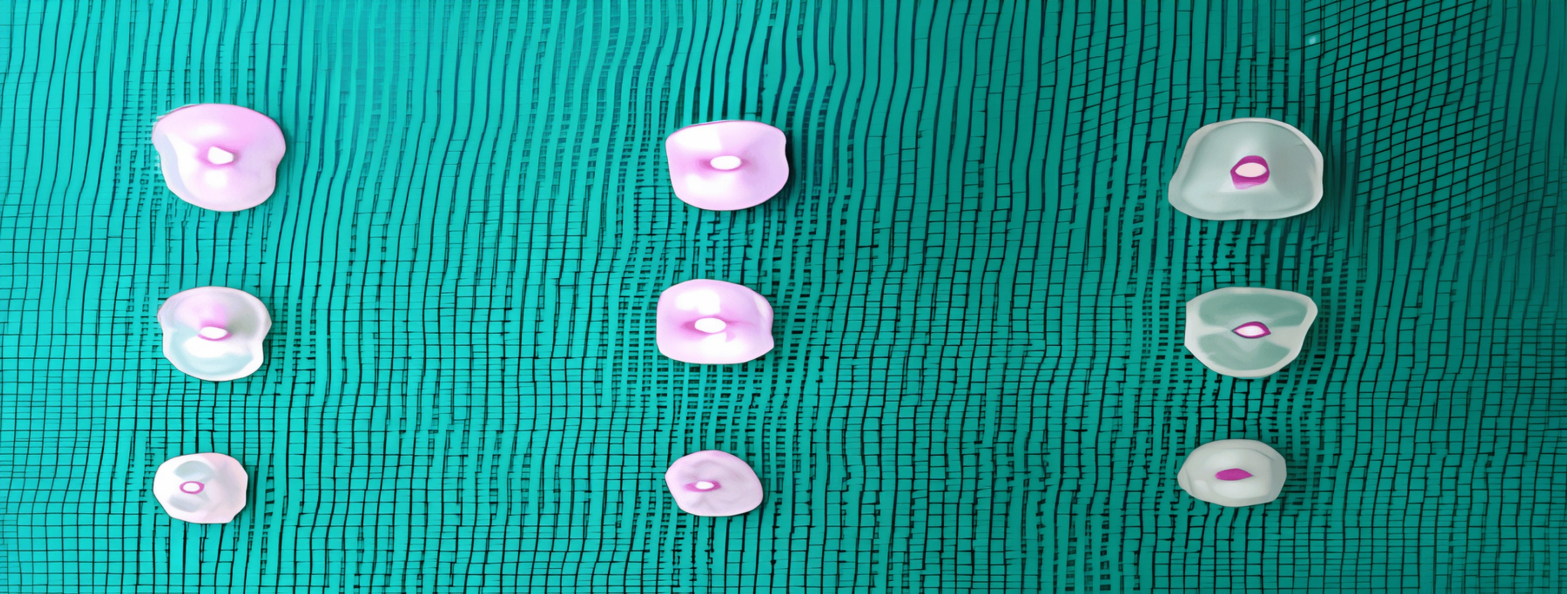
Root sections Obturated tooth samples were sectioned at 6 mm, 4 mm, and 2 mm from the apex to obtain coronal, middle, and apical third sections

For examining the depth of sealer penetration, we sectioned the obturated root samples horizontally at three different levels (i.e., 2 mm, 4 mm, and 6 mm) from the root apex using a hard tissue microtome under copious amounts of water coolant (Figure 1).

Penetration depth measurement

**Figure 2:**
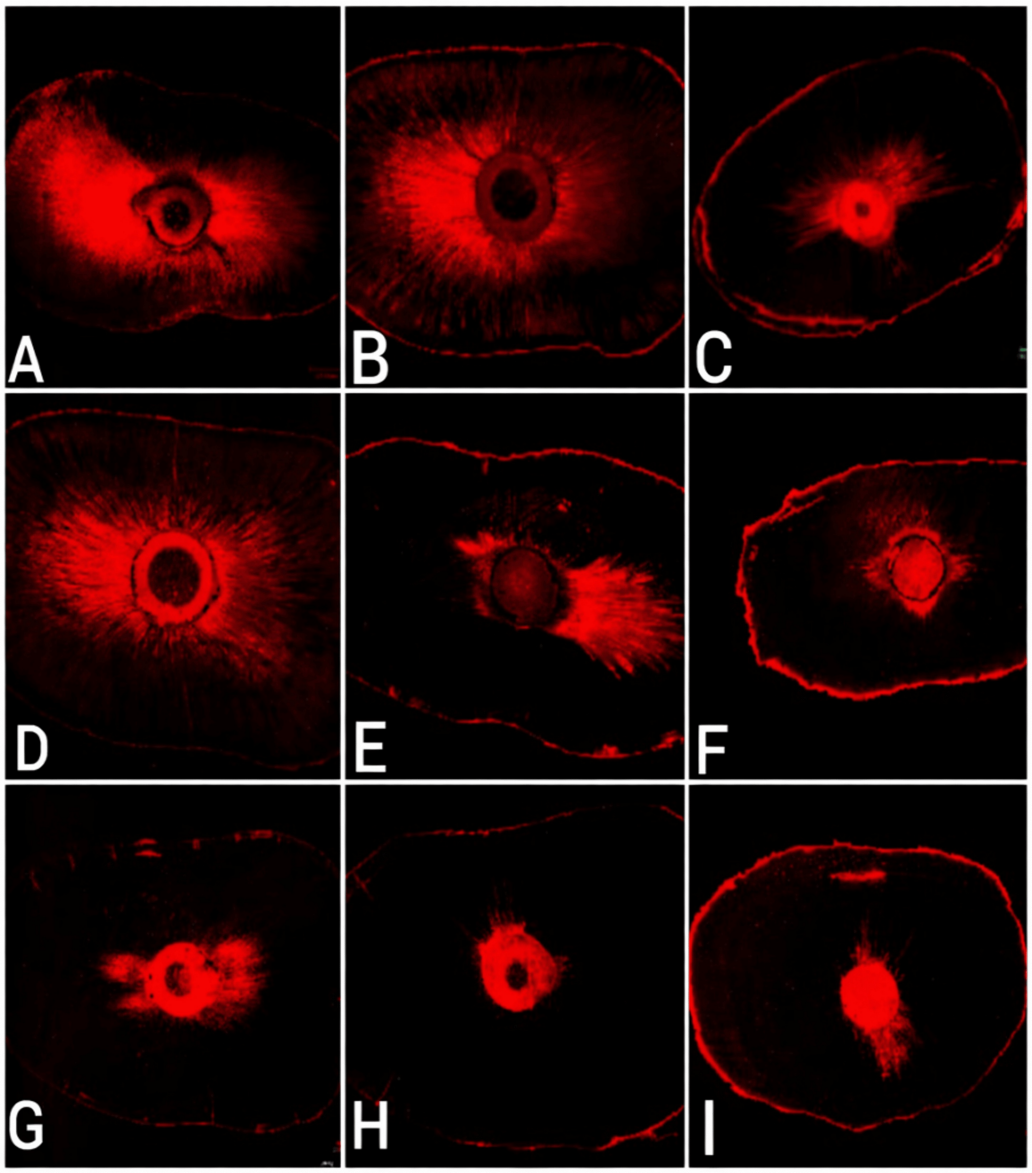
Depth of sealer penetration in the coronal, middle and apical third in the different groups A, B, C: depth of penetration of AH Plus® (Dentsply Sirona Inc., Charlotte, North Carolina, United States); D, E, F: depth of penetration of AHP10; G, H, I: depth of penetration of AHP5 in the coronal, middle and apical third respectively AHP10: AH Plus with a 10:1 ratio of 0.75% pachymic acid; AHP5: AH Plus sealer with a 5:1 ratio of 0.75% pachymic acid

A confocal laser scanning electron microscope (LSM) was used to analyze each specimen. We conducted this investigation at Saveetha Dental College's White Lab in Chennai, Tamil Nadu, India. Adobe Photoshop CS3 (Adobe Inc., San Jose, California, United States) was used to measure the sealer's penetration depth into dentinal tubules. The investigators measured the depth of sealer penetration and recorded it at four standard locations (mesial, distal, buccal, and lingual) on each piece using a rubber tool on the LSM image browser software (Figure 2).

Statistical analysis

Statistical analysis was performed to compare the depth of sealer penetration at the coronal, middle, and apical thirds within each study group. Descriptive statistics were expressed as mean ± standard deviation (SD). One-way analysis of variance (ANOVA) was used to assess differences in mean sealer penetration among the three root levels. A p-value of less than 0.05 was considered statistically significant, corresponding to a 95% confidence level.

## Results

Our team evaluated a total of 30 mandibular premolars for sealer penetration at the coronal, middle, and apical thirds, using confocal laser scanning microscopy. We compared the mean sealer penetration depth among the three groups (A, B, and C) at the coronal, middle, and apical thirds (Table 1). In Group A, the highest mean penetration was observed at the coronal third (1640.80 ± 4.5 µm), followed by the middle third (985.39 ± 3.2 µm) and apical third (387.91 ± 1.9 µm). The difference in penetration depth across the three regions was statistically significant (p = 0.03), indicating that sealer penetration decreased from coronal to apical regions. For Group B, the coronal third showed a mean penetration of 910.04 ± 1.4 µm, the middle third 1007.62 ± 1.9 µm, and the apical third 635.95 ± 2.8 µm. No significant difference was observed between the regions (p = 0.07), suggesting relatively uniform sealer penetration across the root thirds. In Group C, the coronal third penetration was 823.54 ± 3.7 µm, the middle third 334.03 ± 4.6 µm, and the apical third 423.92 ± 2.7 µm. The differences were statistically significant (p = 0.03), showing reduced penetration at the middle third compared to the coronal and apical regions. Overall, Groups A and C demonstrated a significant variation in sealer penetration among coronal, middle, and apical thirds, whereas Group B showed no statistically significant difference. 

**Table 1:** Analysis of variance (ANOVA) was used to compare the differences among the groups p - .0.05- 5% level of significance. * indicates a statistically significant difference among the compared groups

Group		Sealer penetration (µm), mean ± SD	P value
Group A	Coronal	1640.798 ± 4.5	0.03 * (F - 4.588)
	Middle	985.3916 ± 3.2	
	Apical	387.9106 ± 1.9	
Group B	Coronal	910.0442 ± 1.4	0.07 (F-3.302)
	Middle	1007.61867 ± 1.9	
	Apical	635.952 ± 2.8	
Group C	Coronal	823.5386 ± 3.7	0.03 * (F - 4.352)
	Middle	334.0312 ± 4.6	
	Apical	423.9232 ± 2.7	

Table 2 depicts the intragroup comparison of the depth of penetration of Group A (AH Plus sealer) between the coronal middle and apical third and shows a significant difference in penetration depth among the various regions of the root canal (p= 0.012).

**Table 2:** Intragroup comparison of Group A A one-way ANOVA demonstrated a statistically significant difference in the mean depth of sealer penetration among the coronal, middle, and apical thirds in Group A (F(2,11) = 6.848, p = 0.012).

Comparison	Sum of Squares	Df	Mean Square	F	Sig.
Between Groups	3493590	2	1746795	6.848	0.012
Within Groups	2806018	11	255092.5		
Total	6299608	13	-	-	-

Table 3 depicts the intragroup comparison of depth of penetration of Group B (AHP10) in coronal, middle, and apical third, and it is evident that there is no significant difference in penetration depth (p=0.299), showing uniform depth of penetration.

**Table 3:** Intra group comparison of Group B A one-way ANOVA showed no statistically significant difference in the mean depth of sealer penetration among the coronal, middle, and apical thirds in Group B (F(2,13) = 1.325, p = 0.299).

Comparison	Sum of Squares	df	Mean Square	F	Sig.
Between Groups	394241.9	2	197120.9	1.325	0.299
Within Groups	1933749	13	148749.9		
Total	2327991	15	-	-	-

Table 4 depicts the intragroup comparison of the depth of penetration of Group C (AHP5) between the coronal, middle, and apical third and shows that there is a significant difference (p= 0.001) in penetration depth among the various regions of the root canal.

**Table 4:** Intra group comparison of Group C A one-way ANOVA demonstrated a statistically significant difference in the mean depth of sealer penetration among the coronal, middle, and apical thirds in Group C (F(2,13) = 11.257, p = 0.001).

	Sum of Squares	df	Mean Square	F	Sig.
Between Groups	713953.4	2	356976.7	11.257	0.001
Within Groups	412238	13	31710.61		
Total	1126191	15	-	-	-

## Discussion

The long-term success of non-surgical root canal therapy is largely dependent on the hermetic, 3D seal of the root canal spaces. Microorganisms and their byproducts are the primary factors that initiate and maintain periapical inflammation [10]. Achieving a hermetic seal is quite a challenging process and depends on various factors such as proper instrumentation, biomechanical preparation, complete obturation, and post-endodontic restoration.

Gutta-percha, a biocompatible, thermoplastic material, is universally accepted as the “gold standard for obturation" [1]. Though used for ages in dentistry, it has its own disadvantage as it does not adhere to the tooth structure. To overcome this, root canal sealers are designed to fill the voids and irregularities between the dentinal walls and core filling materials [11]. Among the various options of sealers available [4], AH Plus sealer, the popular epoxy resin-based sealer, is considered the gold standard among them. Despite its advantages, the major drawback of AH Plus sealer is its cytotoxicity due to its deleterious components. Research has been carried out perpetually to overcome this cytotoxicity [4,5].

Compared to synthesized medications, which have the significant disadvantage of drug resistance, indigenous phytochemicals have been widely used for their therapeutic qualities, often with fewer adverse effects. Among the various phytochemicals available in the research world, PA is one such phytochemical obtained from *F. nigra* that has antioxidant and anti-inflammatory properties. In line with earlier studies on the modification of AH Plus sealer with phytochemicals [3], a time-dependent increase in cell viability was noticed with the addition of 0.75% pachymic acid in 10:1 and 5:1 ratios. Thus, the same ratios were adapted in our present studies.

The success of root canal treatment depends on the maximum depth of penetration of the sealer into the dentinal tubule, as it influences the sealing ability of obturation by creating an interface with the dentinal tubule, enhancing mechanical interlocking, entombing residual microorganisms, and reducing microleakage. Thus, depth of penetration is considered a critical parameter in evaluating the sealing ability of endodontic sealers. To the best of our knowledge, this is the first study to evaluate the depth of penetration of AH plus sealer modified with PA.

The smear layer plays a vital role in determining the depth of penetration of sealers. It is recommended that NaOCl and chelating agents be used together to remove the smear layer; 5.25% NaOCl and 17% EDTA have been used in the present study to remove the smear layer as per the recommendation [12]. Mandibular premolars with a single root that is 20-22 mm long and has a curvature of less than 5 degrees were chosen for the current investigation in order to reduce morphological diversity and maintain uniformity. According to Wu et al. [13], bucco-lingual and mesio-distal radiographs were used to confirm the existence of a single canal. Confocal LSM was chosen over scanning electron microscope (SEM) to evaluate the depth of penetration of the sealer, as the former facilitates the acquisition of several optical images captured through dentin thickness and can identify fluorescent rhodamine-marked sealers penetrating along the circumference of the canal even at low magnifications such as 50X-1004X [14].

According to the results of the study, all the groups showed reduced sealer penetration in the apical third, consistent with previous reports [15], likely due to fewer and narrower dentinal tubules in this region. The PA-modified AH Plus demonstrated lower penetration in the coronal and middle thirds compared to the conventional sealer, which may be attributed to increased viscosity following modification. These findings agree with Preethi et al. (2020) [5], who reported reduced flow of AH Plus with PA addition within ISO limits. Although the 10:1 ratio showed slightly greater penetration than the 5:1 ratio, the difference was not significant. Clinically, the modified sealer may offer a safer option in wide canals, though further studies are required to enhance penetration and evaluate clinical relevance.

Limitations

A limited sample size and convenient and simple root canal anatomy were the limitations of this study. Only one concentration (0.75%) of PA was evaluated in this study; other concentrations of pachymaic acid are to be verified in future studies.

## Conclusions

Given the constraints of this in vitro investigation, it can be said that the depth of dentinal tubule penetration was affected by the addition of 0.75% PA to AH Plus. In comparison to the adjusted groups, conventional AH Plus showed higher overall penetration. Although the difference was not statistically significant, the 10:1 ratio demonstrated superior penetration compared to the 5:1 ratio. Consequently, PA alteration somewhat decreases sealer penetration depth even though it may have biological benefits. In order to optimize the formulation and assess its long-term clinical efficacy, more research is advised
